# Ultrastructure of cerebral eyes in Oweniidae and Chaetopteridae (Annelida) – implications for the evolution of eyes in Annelida

**DOI:** 10.1186/s40851-022-00188-0

**Published:** 2022-01-25

**Authors:** Günter Purschke, Stepan Vodopyanov, Anjilie Baller, Tim von Palubitzki, Thomas Bartolomaeus, Patrick Beckers

**Affiliations:** 1 Zoology and Developmental Biology, Department of Biology and Chemistry, Osnabrück University, Osnabrück, Germany; 2 Department of Invertebrate Zoology, Biological Faculty, Lomonosov Moscow State University, Moscow, Russia; 3 Present address: Department of Biology, Faculty II, University of Vechta, Vechta, Germany; 4 Institute of Evolutionary Biology and Ecology, University of Bonn, Bonn, Germany

**Keywords:** Annelida, Palaeoannelida, Chaetopteriformia, Polychaetes, Rhabdomeric photoreceptor cell, Ciliary photoreceptor cell, Pigment cell, Pigment cup eye, Pigment spot eye

## Abstract

**Background:**

Recent phylogenomic studies have revealed a robust, new hypothesis of annelid phylogeny. Most surprisingly, a few early branching lineages formed a basal grade, whereas the majority of taxa were categorized as monophyletic Pleistoannelida. Members of these basal groups show a comparatively simple organization lacking certain characters regarded to be annelid specific. Thus, the evolution of organ systems and the characteristics probably present in the last common annelid ancestor require reevaluation. With respect to light-sensitive organs, a pair of simple larval eyes is regarded as being present in their last common ancestor. However, the evolutionary origin and structure of adult eyes remain obscure. Typically, adult eyes are multicellular pigment cups or pinhole eyes with or without a lens comprising rhabdomeric photoreceptor cells (PRCs) and pigmented supportive cells (PSCs) in converse design. However, in the most basal lineages, eyes are only present in a few taxa, and thus far, their ultrastructure is unknown.

**Results:**

Ultrastructural investigations of members of Oweniidae and Chaetopteridae reveal a corresponding design of adult cerebral eyes and PRCs. The eyes in species of these groups are simple pigment spot eyes, either forming a flat patch or embedded in a tube-like invagination. They are part of the epidermis and comprise two cell types, PSCs and rhabdomeric PRCs. Both cell types bear microvilli and one more or less reduced cilium. However, the PRCs showed only a moderate increase in the apical membrane surface in the form of irregularly arranged microvilli intermingling with those of the PSCs; a densely arranged brush border of rhabdomeric microvilli was absent. Additionally, both cell types show certain characteristics elsewhere observable in typical epidermal supportive cells.

**Conclusions:**

These findings shed new light on the evolutionary history of adult eyes in Annelida. Most likely, the adult eye of the annelid stem species was a pair of simple pigment spot eyes with only slightly specialized PSCs and PRCs being an integrative part of the epidermis. As is the case for the nuchal organs, typical pigment cup adult eyes presumably evolved later in the annelid phylogeny, namely, in the stem lineages of Amphinomida and Pleistoannelida.

## Background

The monophyly and taxonomic composition of Annelida have been debated for decades [1]. These controversies have been based on opposing morphological hypotheses, and until the end of the first decade of this century, analyses based on molecular data could not resolve these discrepancies, leaving annelid phylogeny unresolved [1–5]. However, these problems seem to be solved with current phylogenomic data hypotheses, resulting in a more or less widely accepted robust phylogenetic hypothesis [6–14]. Although many annelid taxa have still not been included, the incorporation of missing taxa did not substantially change the so-called backbone of the annelid tree [1, 8].

**Fig. 1:**
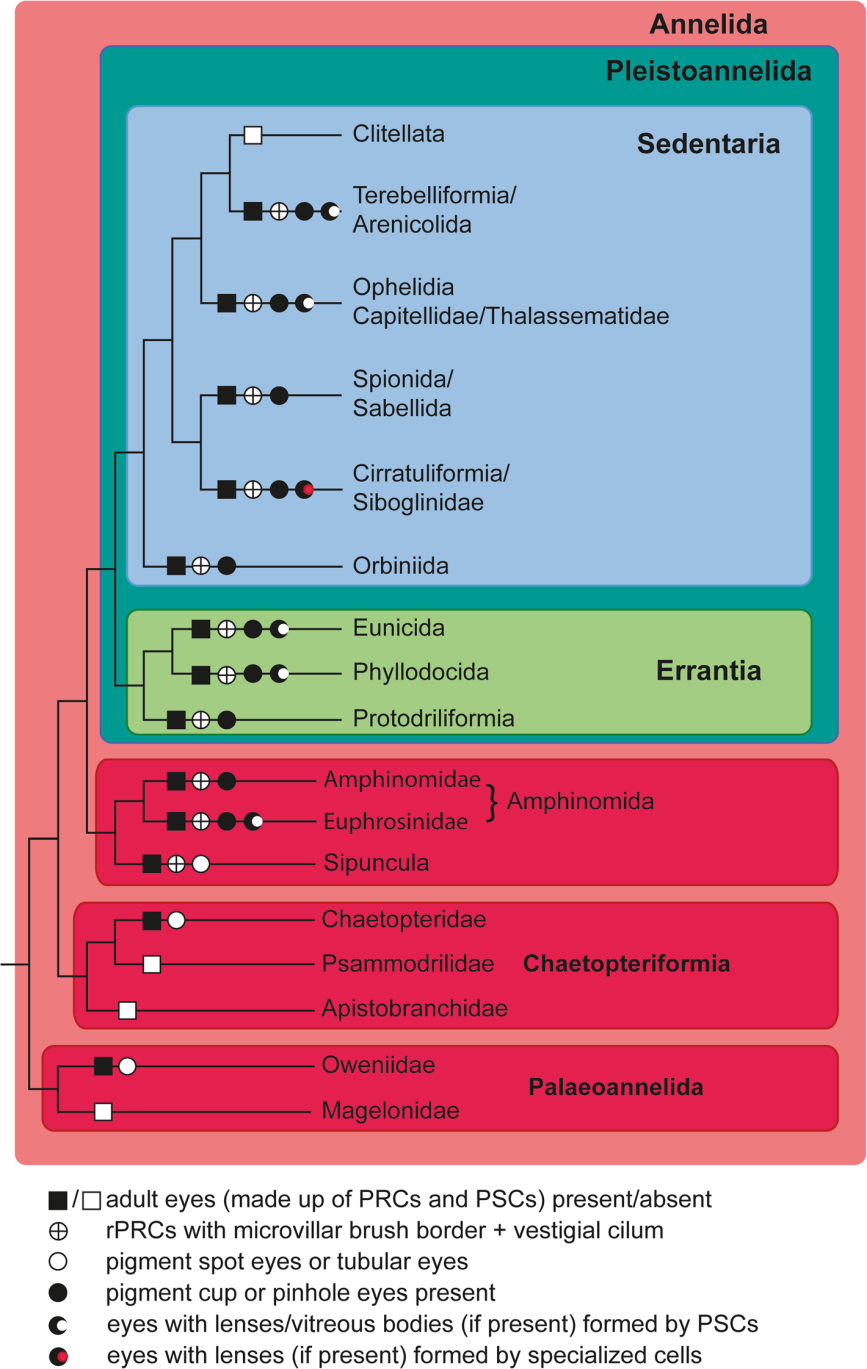
Simplified phylogenetic tree of the major annelid taxa based on recent phylogenomic studies [1, 6–15]. In the lineages leading to terminals, characteristics of adult cerebral eyes are included [16–22]. Note that a given character state is not necessarily present in every member or subgroup of this clade

This revised phylogeny partially led to a new and somehow unexpected order of clades within Annelida. In these analyses, there was consistently a basal grade of three taxa, Palaeoannelida, Chaetopteriformia, and Sipuncula plus Amphinomida, all of which were formerly placed elsewhere in the annelid tree [1, 8]. The vast majority of taxa fall into Errantia or Sedentaria, united as Pleistoannelida (Fig. 1). Members of the basal branching taxa are characterized by a comparatively simple organization and lack certain characteristics formerly regarded as typical for annelids. These recent changes prompted a discussion about the characteristics of the last common ancestor of annelids. They also initiated a series of character analyses in these basal lineages to shed light on character evolution within Annelida [11, 15, 16, 23, 24].

Although widespread in annelids, eyes have not been included in such investigations, except for some preliminary observations in *Owenia fusiformis* [15]. Due to their common occurrence within the group and their striking diversity in terms of structure and numbers, the morphology of these organs may serve as an ideal candidate for elucidating their evolutionary history [5]. Eyes in Annelida may be cerebral or occur elsewhere on the body, the latter commonly called ectopic eyes [5, 17–19]. Regarding the former, larval and adult eyes are generally distinguished by differing structures and molecular fingerprints. Although not in the focus of this review, the annelid stem species must have possessed a pair of simple larval eyes involved in phototaxis and directional photoreception [8, 25, 26].

Despite this knowledge, the evolutionary origin and history of adult eyes have not thus far been resolved. These eyes are pigment cup or pinhole camera eyes equipped with sophisticated photoreceptor cells (PRCs) of the rhabdomeric type and pigmented supportive cells (PSCs) [5, 18–20]. The highest degree of complexity occurs in members of the more vagile and sometimes predatory forms in the clade Errantia (Fig. 1). Especially in this group, additional light guiding structures, either termed lenses or vitreous bodies, may also be present [17–19]. Since the discovery of such highly complex eyes in certain groups of Sedentaria (e.g., Orbiniidae and Flabelligeridae), these eyes are regarded as already being present in the stem lineage of Pleistoannelida, although most Sedentaria possess simple eyes of the larval type [21]. However, since two pairs of similar eyes also occur in Amphinomida, the sister group of Pleistoannelida, the most parsimonious hypothesis is a single origin of two pairs of adult pigment cup eyes in the stem lineage of these two clades or even earlier [20–22]. To test this hypothesis, we studied the anatomy of the adult eyes in the remaining taxa Palaeoannelida comprising Oweniidae and Magelonidae, as well as Chaetopteriformia sensu Helm et al. [11] with Chaetopteridae, Psammodrilidae, and Apistobranchidae.

Out of the five taxa in these basal lineages, pigmented adult eyes are only known in two taxa, one in each clade: Oweniidae and Chaetopteridae. Other members of the two clades were also included to confirm the absence of pigmented eyes or vestiges thereof in the remaining taxa. Finally, we investigated the ultrastructure of the adult eyes in two species of each group: *Owenia fusiformis* and *Galathowenia oculata* as well as *Spiochaetopterus costarum* and *Phyllochaetopterus socialis.* Without exception, there is only one pair of eyes in these species exhibiting a diverging and, on first observation, puzzling ultrastructure. As a result, we can now propose a modified and more reliable hypothesis on the evolution of adult eyes in Annelida, which is congruent with recent phylogenetic hypotheses. We provide evidence for the presence of a pair of simple eyespots in the last common ancestor of Annelida. These eyes are not distinctly set off from the surrounding epidermis, exhibiting only moderately specialized receptor and pigment cell morphology. From their structure, they are presumed to be capable of directional photoreception only [27, 28]. This report represents the first description of such simple structured eyes in Annelida.

## Results

### Position and external structure of eyes

**Fig. 2:**
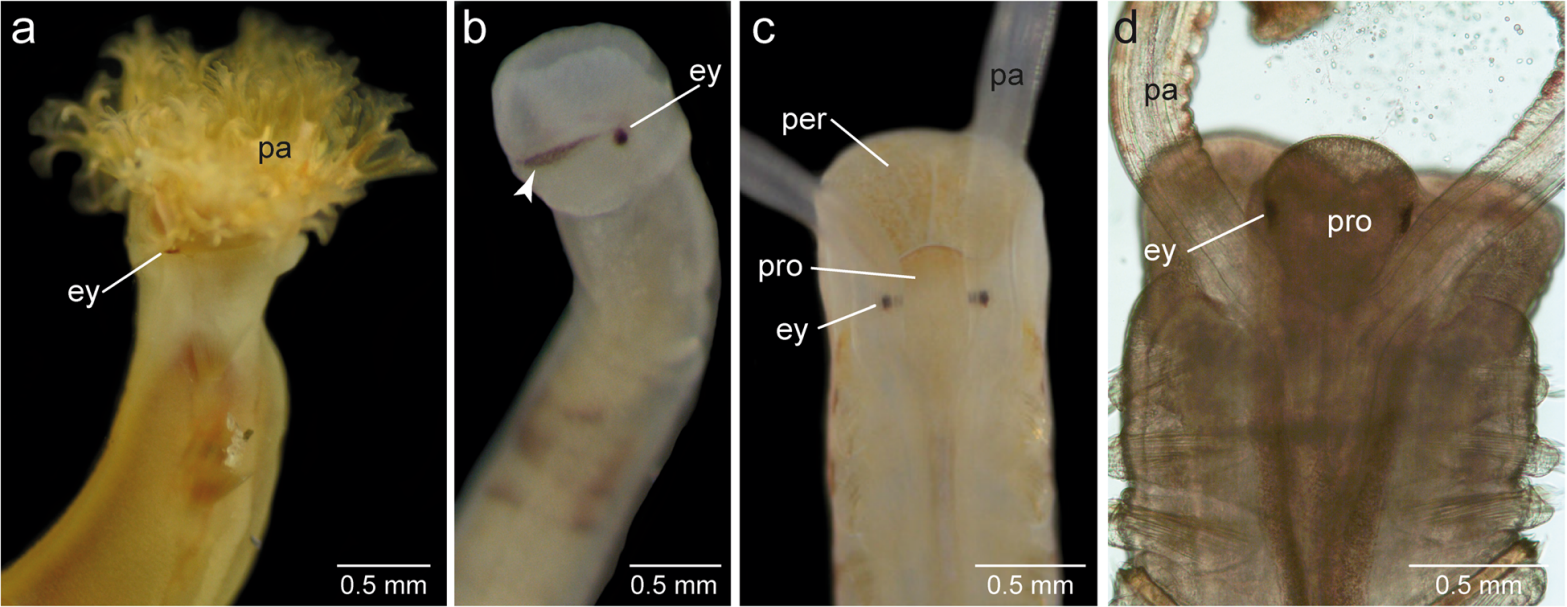
Anterior ends of species investigated and position of cerebral eyes (ey). Micrographs of living individuals or specimens **a)** *Owenia fusiformis*, **b)** *Galathowenia oculata*; note the additional transverse dorsolateral band of pigments (arrowhead), **c)** *Spiochaetopterus costarum,* and **d)** *Phyllochaetopterus socialis.* **a, b**, lateral view, **c, d,** dorsal view. *Abbreviations: ey eye, pa palp, per peristomium, pro prostomium*

Externally visible reddish or black pigment spots are present on the anterior ends in *Owenia fusiformis, Galathowenia oculata, Chaetopterus norvegicus, Spiochaetopterus costarum*, and *Phyllochaetopterus socialis.* Generally, these structures are regarded to represent eyes. Such externally visible pigment spots are absent in adult *Magelona mirabilis, Psammodrilus balanoglossoides*, and *Apistobranchus typicus.* In *O. fusiformis,* these eye spots are reddish and ovoid, and their long axis is oriented transversely and situated just beneath the tentacular crown (Fig. 2a). The black eyes of *G. oculata* are roundish and situated ventrolaterally in the middle of the truncated head region (fused pro- and peristomium, see Parapar et al. [29]), posterior to the slit-like mouth (Fig. 2b). Two long transverse eyebrow-like pigment bands accompany the eyes in this species. These bands run transversely and taper towards the dorsal midline, leaving a small gap in the median. In *C. norvegicus, S. costarum*, and *P. socialis*, the roundish black eyes were more distinct and situated laterally on the prostomium (Fig. 2c, d). In these species, the prostomium is a small lobe situated dorsally above the collar-like peristomium and laterally flanked by the palps.

### The general structure of eyes

**Fig. 3:**
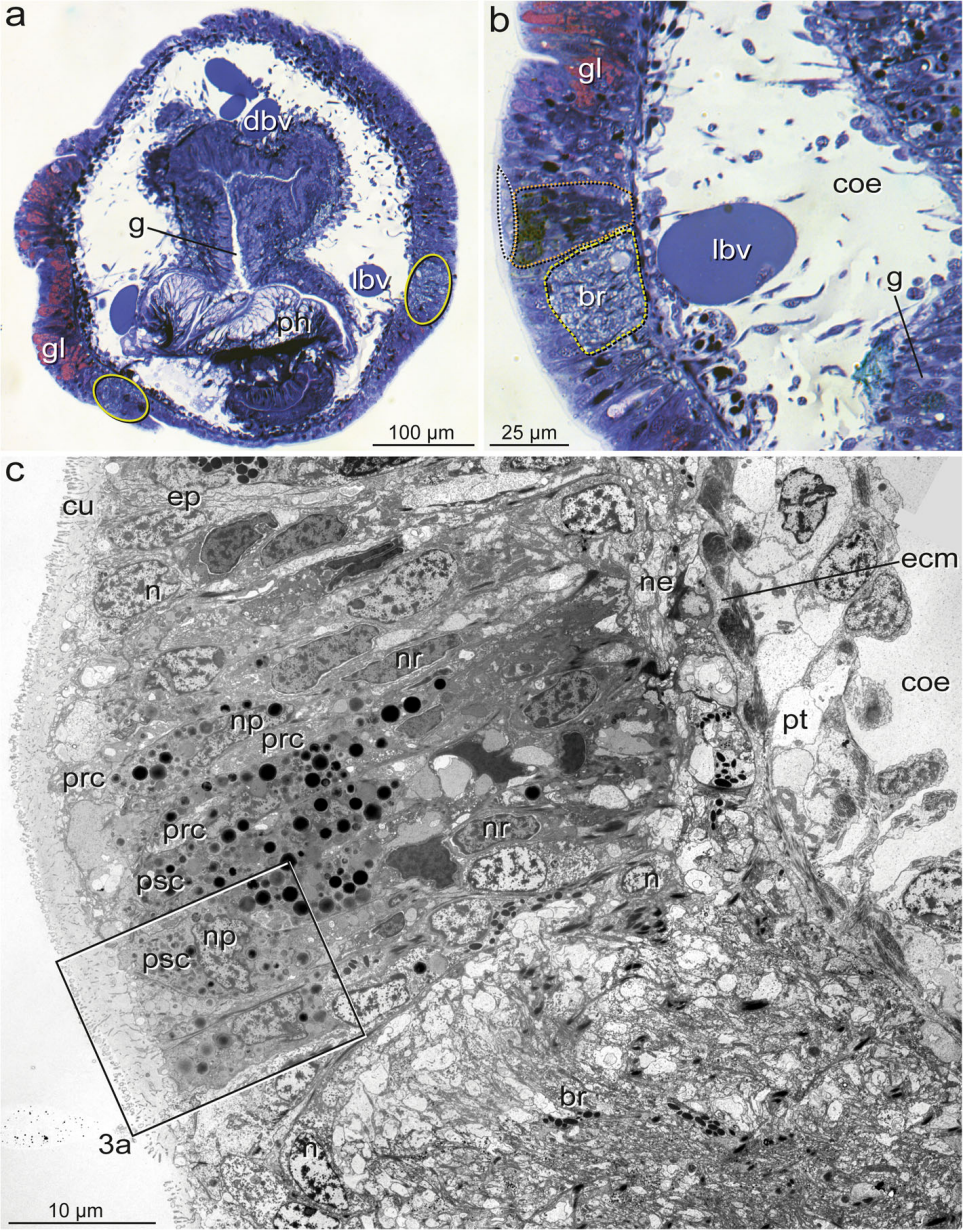
*Galathowenia oculata.* The general structure of cerebral eyes. **a, b)** LM of semi-thin sections, **c)** TEM; **a)** Cross-section behind the mouth and pigmented eyes spots, note the lateral position of the ring-like brain inside the epidermis (encircled), on the left epidermis with numerous gland cells (gl, stained in pink); **b**) Brain (br, encircled in yellow) with an adjacent eye (ey, encircled in red) located dorsally, eye not distinctly set off from surrounding tissue, pigment cells leave an upper, biconvex lens-shaped part without pigment (encircled in white); **c)** TEM of the ultrathin section adjacent to **b** with PSCs (psc) and PRCs (prc), nuclei (np, nr) arranged in two layers: those of PSCs (np) above those of PRCs (nr), basal region of the eye with a network of neurites (ne), boxed area enlarged in **3a**. *Abbreviations: br brain, coe coelom, cu cuticle, dbv dorsal blood vessel, ecm ECM, ep epidermis, gl glandular cell, g gut, lbv lateral blood vessel, n nucleus, ne neurite, np nucleus of PSC, nr nucleus of PRC, ph pharynx, prc PRC, psc PSC, pt peritoneum*

In *Owenia fusiformis* and *Galathowenia oculata,* eyes are represented by flat pigment spots. These spots are continuous with the epidermal epithelium and not distinctly set off from the surrounding cells (Figs. 3a-c, 6a). In *Chaetopterus norvegicus, Spiochaetopterus costarum*, and *Phyllochaetopterus socialis*, the eyes lie in tube-like or slit-like invaginations, either laterally on the flat dorsum of the prostomium (*S. costarum,* approximately 150 μm deep, Fig. 7a, b) or laterally between the prostomial lobe and the peristomium (*P. socialis,* approximately 30 μm deep, Fig. 8a, b). The epithelium (retina) forming the eye proper was concentrated on the outwards facing side of the tube, extending into the bottom in *S. costarum* (Fig. 7a, b). In contrast, in *P. socialis*, the retina does not include the bottom of the invaginations and has the shape of a laterally oriented pigment spot (Fig. 8a, b). In *S. costarum* and *P. socialis*, the remaining sides of the invaginations opposite to the retinae are formed by regular epidermal cells. The invaginations are filled with cuticular material or processes of the cells forming the invaginations (Figs. 7a, b, 8b, c, 9a).

In all species, the eyes consist of only two cell types: pigmented supportive cells (PSCs) and photoreceptor cells (PRCs). The epidermis forms a comparatively thick epithelium in the head region and is composed of columnar cells (Figs. 3a, b, 8a). It is approximately 45 μm thick in the eye region of *G. oculata,* up to 80 μm in *O. fusiformis,* 40–60 μm in *P. socialis*, and 50–150 μm in *S. costarum.* In the latter two species, the epidermis in the eye region diminishes in thickness by approximately 20 μm and 80 μm, respectively, from dorsal to ventral (Figs. 7a, 8b, c). In the lower third, approximately 17 μm from the bottom of the fold, the epithelium forming the eye exhibits a small fold up to 9 μm deep in *P. socialis* (Fig. 8b, c). The nuclei are staggered in the apical-basal direction, giving the epithelium a pseudostratified appearance in all species (Fig. 3c). The epidermis is rich in glandular cells, which extend close to the eyes proper (e.g., Figs. 3a, b, 8a-c).

The eyes are situated in close vicinity of the ring-like brains. They either lie directly above the neuropil of the brain (*O. fusiformis, S. costarum, P. socialis;* Figs. 6a, 7a, 8b, c) or are situated somewhat dorsally above the neuropil (*G. oculata;* Fig. 3b, c). The pigment granules are more or less irregularly distributed in the eye region. However, they mostly accumulate in the upper half of the epithelium, whereas some pigment granules are present basally in the oweniid species (Figs. 3b, c, 6a). In the eyes of *O. fusiformis*, certain PSC processes containing pigment granules partly envelope the brain’s neurons (Fig. 6a, b). In this species, pigment granules are more irregularly distributed throughout the entire eye region and not as densely arranged as in the other three species. Pigment granules accumulate apically in the eye region with some pigment-containing basal processes separating groups of cell bodies in *P. socialis* (Fig. 8c). In *S. costarum*, the eyes are more distinctly set off and characterized by a high pigment content (Fig. 7a, b). Their pigment content decreases distally. Analyses of either histological *(Magelona mirabilis, Apistobranchus typicus)* or ultrathin (*Psammodrilus balanoglossoides*) serial sections revealed neither small pigmented nor unpigmented photoreceptor–like structures beyond those already known [19, 30–32].

### The pigmented supportive cells (PSCs)

**Fig. 4:**
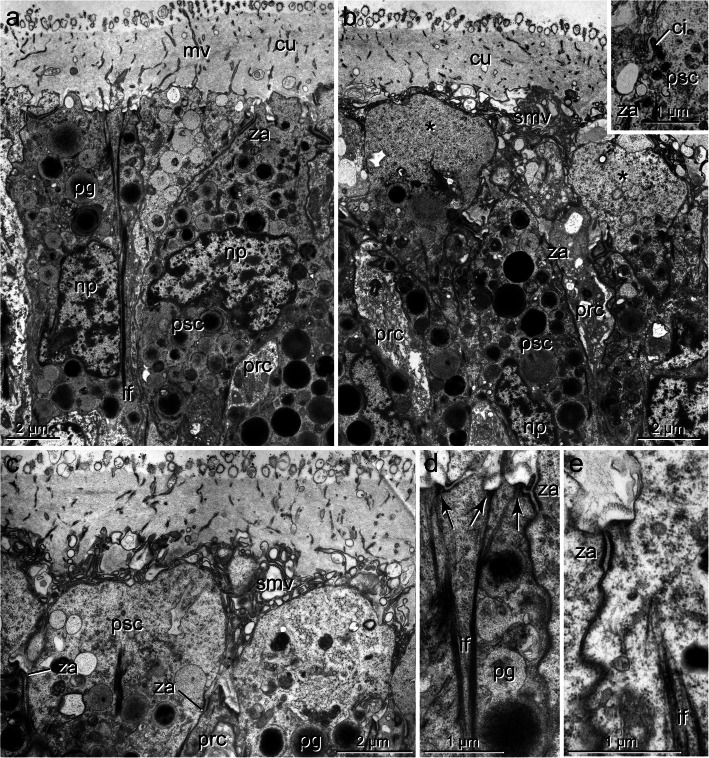
*Galathowenia oculata.* Cerebral eye, PSCs. TEM. **a)** Enlargement of the boxed area from Fig. **3****c**, ventral group of PSCs (psc) facing the brain with densely packed pigment granules (pg) extending to the apical membrane; **b)** Medial and dorsal group of PSCs (psc) with pigment-free bubble-like apices (asterisks), processes of PRCs (prc) terminating between apices of PSCs; **inset:** apex of PSC with residual cilium (ci); **c)** Apices of dorsal PSCs, bubble-like swellings extend above the level of junctional complexes (za), sensory microvilli (smv) form layer above and between PSCs apices; **d, e)** Apical regions of PSCs with bundles of intermediate filaments (if) terminating in small hemidesmosomes (arrows). *Abbreviations: ci cilium of PSC, cu cuticle, if intermediate filaments, mv microvillus, np nucleus of PSC, pg pigment granule, prc PRC, psc PSC, smv sensory microvilli, za zonula adherens*

**Fig. 5:**
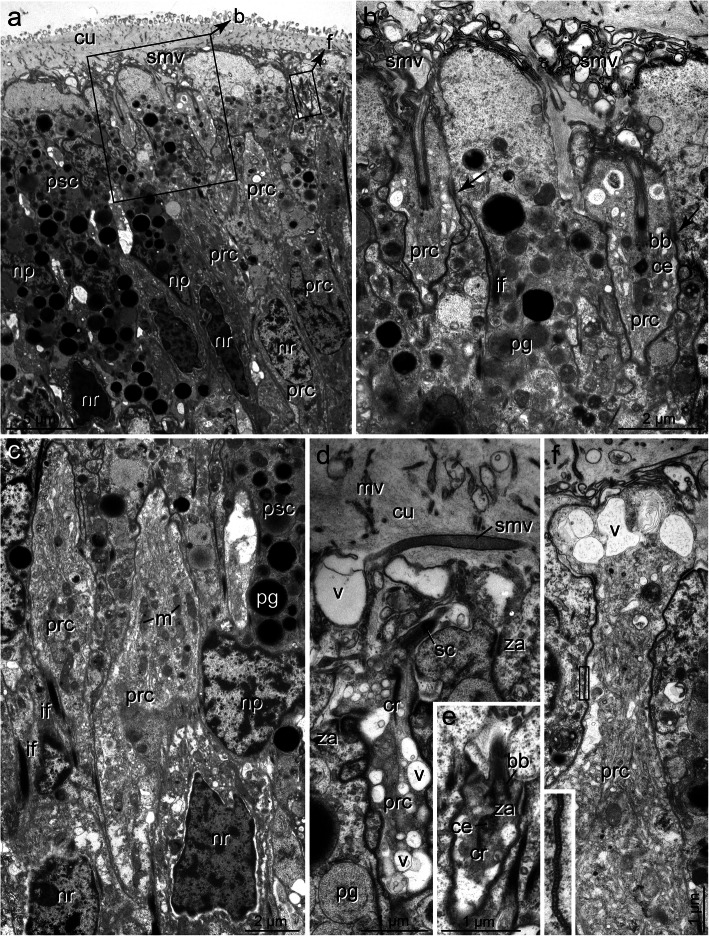
*Galathowenia oculata.* Cerebral eye, PRCs. TEM. **a)** Eye with ciliated PRCs (prc, boxed) between PSCs (psc). Somata of PRCs below cell bodies of PSCs; **b)** enlargement of left boxed area from **a**, two sensory dendrites with cilia (sc) emerging from apical depression of receptor cells, apical regions of PSCs are pigment-free, arrows point to junctional complexes; **c)** Somata of adjacent PRCs (prc) with numerous endo-membranes and mitochondria (m); **d),** Apex of PRC (prc), dendritic process with cilium and microvillus, note electron-lucent vesicles (v); **e)** Sensory cilium with the basal body (bb), accessory centriole (ce) and small rootlet (cr); **f)**, Periphery of PRC process with numerous electron vesicles (v) and agranular ER, inset: enlargement of the boxed area with septate junction. *Abbreviations: bb basal body, ce accessory centriole, cr ciliary rootlet, cu cuticle, if intermediate filaments, m mitochondrion, mv microvillus, np nucleus of PSC, nr nucleus of PRC, pg pigment granule, prc PRC psc PSC, sc sensory cilium, smv sensory microvilli, v vesicle, za zonula adherens*

**Fig. 6:**
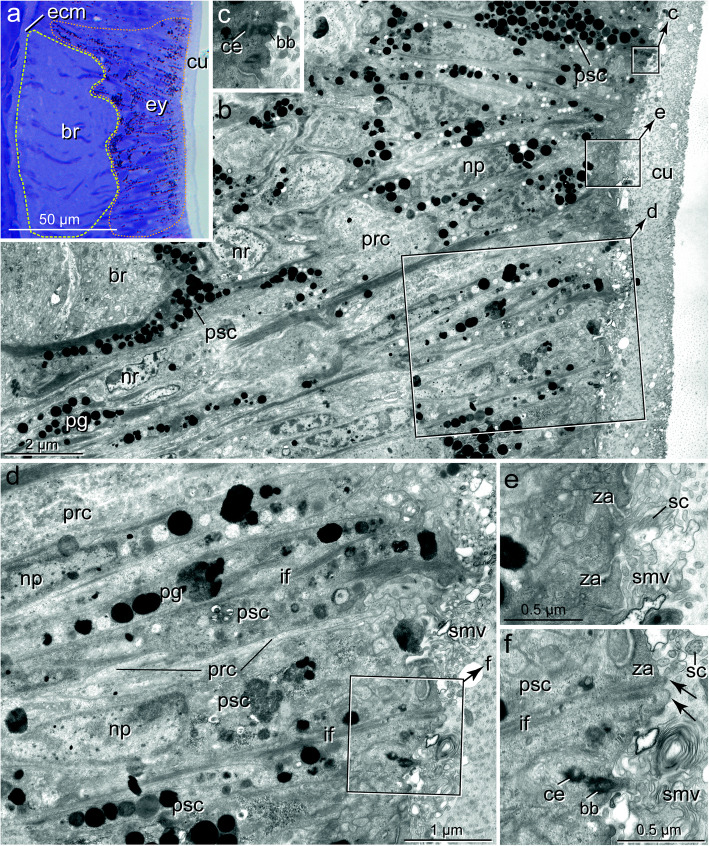
*Owenia fusiformis.* Cerebral eye, **a)** LM, semi-thin section, **b-f)** TEM; **a)** Eye (ey encircled, orange) above brain (br, encircled, yellow), eye not distinctly set off from surrounding tissues, brain traversed by processes of glial cells, visible as basal-apical dark strands; **b)** TEM overview of region similar to **a;** PSCs (psc) and PRCs (prc) intermingle and are hard to discriminate, pigment granules (pg) randomly distributed over the entire eye region. Nuclei of PSCs (np) situated above nuclei of PRCs (nr), note bundles of intermediate filaments (if) in PSCs, boxed areas indicate the position of magnifications in **c, d,** and **e**, **c)** magnification of uppermost box with centriole (ce) and basal body (bb); **d)** Enlargement of **b** with alternating PSCs (psc) and PRCs (prc), eye epithelium covered by irregular arranged sensory processes (smv), the boxed area indicates an enlarged area of **f; e)** Apex of PRC, **f)** Apex of PSC (psc) with accessory centriole (ce) and basal body (bb) of the small cilium, Arrows point to apical hemidesmosomes. *Abbreviations: bb basal body, br brain, ce accessory centriole, cu cuticle, ecm ECM, ey eye, if intermediate filaments, np nucleus of PSC, nr nucleus of PRC, pg pigment granule, prc PRC, psc PSC, sc sensory cilium, smv sensory microvilli, za zonula adherens*

**Fig. 7:**
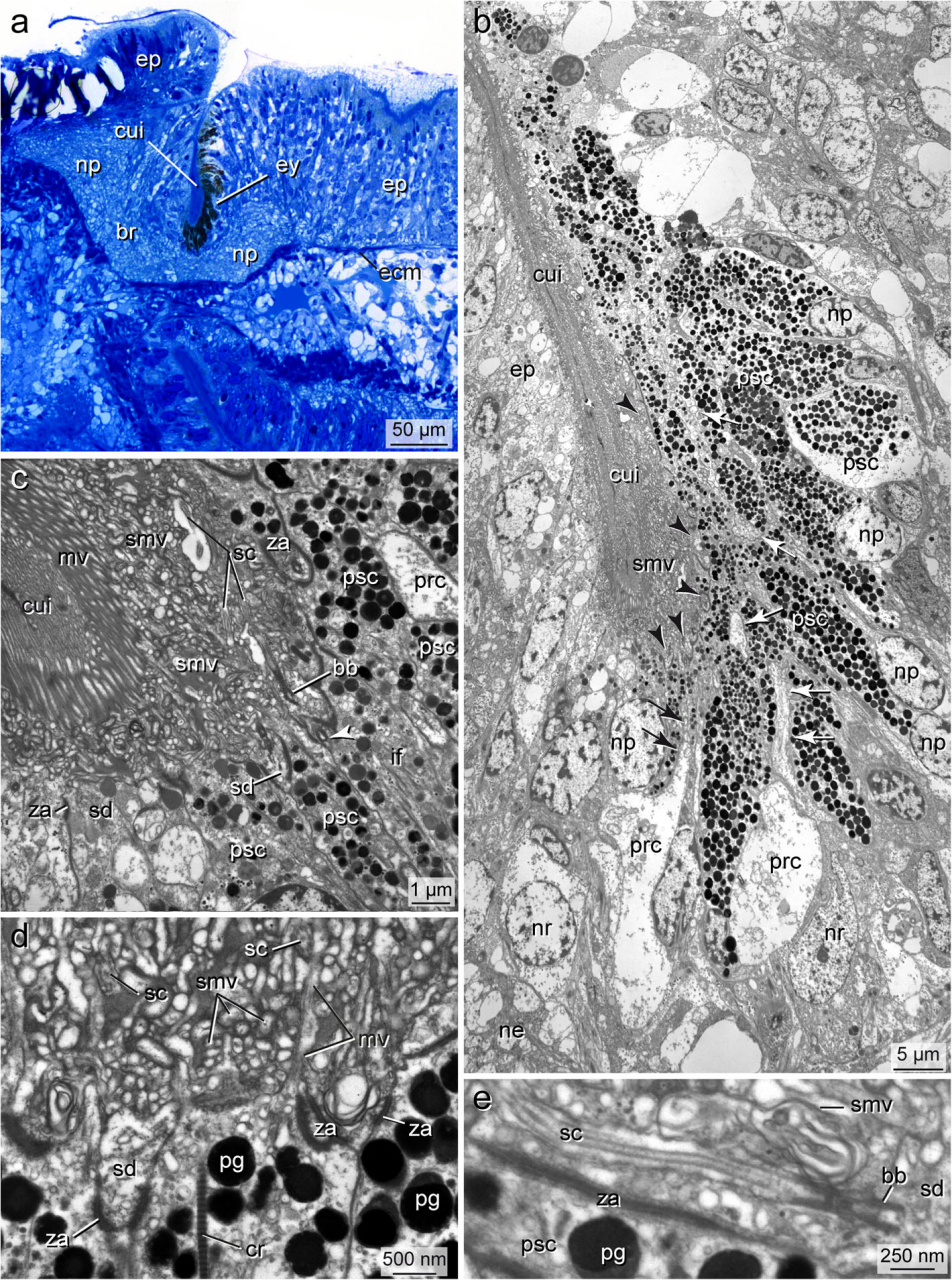
*Spiochaetopterus costarum,* cerebral eye, a LM, semithin section, b-e, TEM; **a)** Part of cross section with left eye (ey) situated in a tube-like invagination filled with cuticle (cui) and extending down to the neuropil (np) of the brain (br), pigment cells form flat layer of medial side; **b)** Low power TEM micrograph showing the eye in longitudinal section, retina composed of numerous PSCs (psc) between which thin processes of PRCs running apically (white arrows), these terminate apically between PSCs (black arrowheads), cuticular invagination (cui) occupied by numerous cell processes (smv); **c)** Bottom of invagination with basal part of retina at higher magnification, monociliary sensory dendrites (sd) of PRCs terminate between PSCs (psc), invagination (cui) basally occupied by sensory cilia (sc),sensory microvilli (smv) and microvilli of PSCs, the latter forming apical layer of parallel microvilli (mv), arrowhead points to basal body in PSC; **d)** Intermingling processes of PRCs and PSCs; **e)** Higher magnification of sensory dendrite (sd) with sensory cilium (sc). *Abbreviations: bb basal body, br brain, cui cuticular invagination, ecm ECM, ep epidermis, ey eye, if intermediate filaments, mv microvillus of PSC, ne neurite, np nucleus of PSC, nr nucleus of PRC, pg pigment granule, prc PRC, psc PSC, sc sensory cilium, sd: sensory dendrite, smv sensory microvilli, za zonula adherens*

**Fig. 8:**
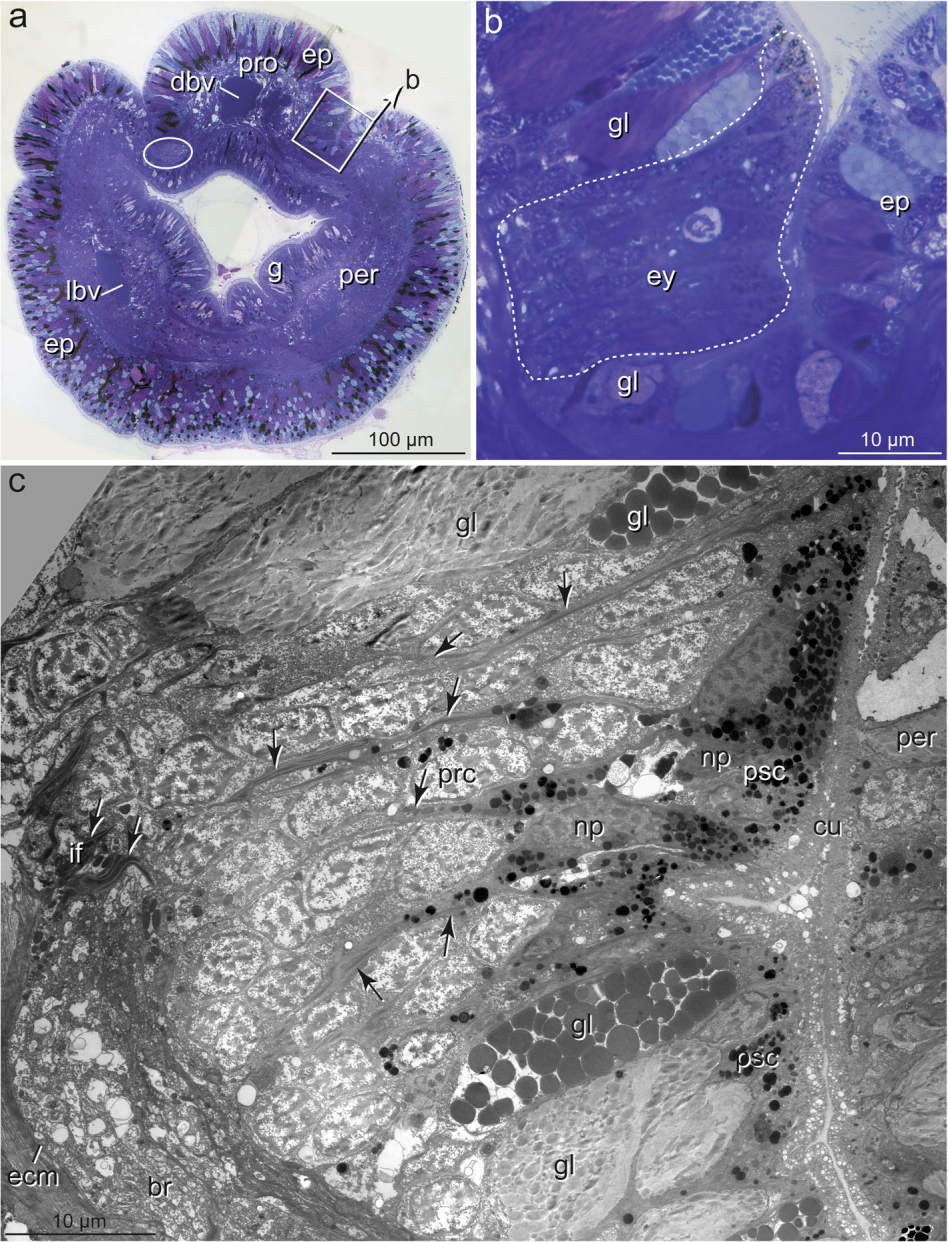
*Phyllochaetopterus socialis,* cerebral eye, **a, b)** LM, semithin sections, **c)** TEM; **a)** Cross-section with the posterior part of prostomium (pro) lying above peristomium (per), eye situated in an invagination between pro- and peristomium (boxed and enlarged in **b**), epidermis rich in glandular cells, ring-like brain encircled; **b)** Enlargement of **a**, eye (ey, encircled by stippled line) with irregular and diffuse outline; **c)** Low power TEM micrograph of the section adjacent to **b**, the entire eye with PSCs (psc) and PRCs (prc), nuclei of PSCs (np) situated above somata of PRCs (nr), arrows point to processes of PSCs extending basally towards ECM. *Abbreviations: br brain, cu cuticle, dbv dorsal blood vessel, ep epidermis, ey eye, g gut, gl glandular cell, if intermediate filaments, lbv lateral blood vessel, np nucleus of PSC, nr nucleus of PRC, per peristomium, pro prostomium, prc PRC, psc PSC*

**Fig. 9:**
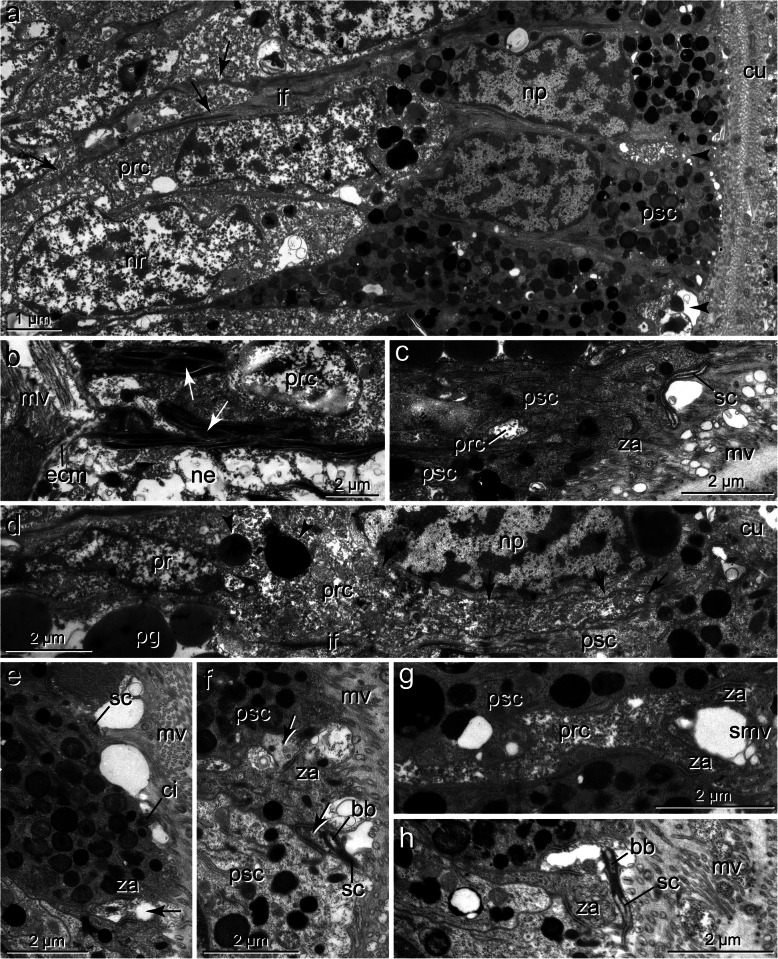
*Phyllochaetopterus socialis.* Cerebral eye, TEM; **a)** PSCs (psc) above group of PRC somata (prc), arrows point to basal extensions of PSCs containing bundles of intermediate filaments running between somata of PRCs, arrowheads: apices of PRCs; **b)** Basal processes of PSCs with densely arranged intermediate filaments (arrows) approaching ECM (ecm), **c)** Apical branching pattern of intermediate filaments entering microvillar bases; **d)** Soma of PRC sending dendritic process apically (arrows), PRC with a few pigment granules (arrowhead); **e)** PSC with apical microvilli (mv) and cilium (ci), arrow: PRC process; **f)** PSCs and two processes of PRCs (arrows), one of which with sensory cilium (sc); **g)** PRC process with apical depression and sensory microvilli (smv); **h)** Sensory cilium emerging from PRC. *Abbreviations: bb basal body, ci cilium of PSC, cu cuticle, ecm ECM, if intermediate filaments, mu muscle fiber, mv microvillus, ne neurites, np nucleus of PSC, nr nucleus of PRC, pg pigment granule, prc PRC, psc PSC, sc sensory cilium, smv sensory microvillus, za zonula adherens*

The PSCs form the major part of the apical region in the eyes of all species studied; their apices are a direct continuation of the surface of the adjacent epidermal cells (Figs. 3b, c, 6a, 7b, 8a, b). These cells are an integrative part of the epithelium and accordingly are connected to their neighbors by zonulae adherents followed by septate junctions (exemplified in Figs. 4e, 5f). The most characteristic feature is their densely packed pigment granules, which accumulate in the upper part of the eyes (Figs. 3c, 4a, b, 5a, b, 6b, d, 7a-e, 8c, 9a, e). The pigment granules are membrane-bound; in *G. oculata*, they measure up to 1.3 μm in diameter, 0.7 μm in *O. fusiformis*, and 0.6 μm in *S. costarum* and *P. socialis* (*n* = 10 each). In *G. oculata*, pigment granules are mainly found in the upper half of the epithelium. In contrast, in *O. fusiformis*, they extend deeper into the PSCs, and in *P. socialis*, most granules are present in the upper quarter, with only some granules occurring in the basal processes (Fig. 8c). In *S. costarum*, the granules form a thick apical layer at least 10 μm thick, extending up to 25 μm (Fig. 7b). In *O. fusiformis*, the pigment content appears completely electron-dense and homogeneous, whereas in *G. oculata, S. costarum*, and *P. socialis*, a certain part of these granules is less dense and more or less electron-dense, sometimes with granular contents.

The cell bodies of the PSCs were 4–7 μm wide (*n* = 8, each) except for the cells of *S. costarum*, which have somata up to 10 μm wide. The nuclei are situated at least 6 μm underneath the epithelial surface (Figs. 3c, 4a). They belong to the region with pigment granules. In *S. costarum*, the nuclei are located at the bottom or below the pigment layer (Fig. 7b). In *G. oculata*, they are arranged in two more or less distinct layers; the second layer of PSC nuclei terminates approximately 20 μm below the epithelial surface (Fig. 3c). In this species, the nuclei have an irregular outline with spherical depressions of housing pigment granules, whereas this feature is less obvious or absent in the other three species.

Only in *G. oculata *﻿can two types of PSCs be distinguished by the different distribution of their pigment granules. In a ventral line comprising two to three rows of cells closest to the brain, the pigment granules extend up to the apices (Figs. 3c, 4a). In the dorsally adjacent PSCs, the apical part is pigment-free (Figs. 3c, 4b, c). These pigment-free parts form somewhat ovoid extensions up to 5.5 μm wide and 3.5 μm high. These extensions arise above the level of the junctional complexes, which occur below the pigment-free apical parts in this region (Figs. 4b, c, 5a, b). This pigment-free area is visible in semithin sections as a flat biconvex lens-like structure above the pigmented region (Fig. 3b, stippled in white).

Apically, the PSCs are covered by a cuticle structurally similar to that of the adjacent epidermal regions (Figs. 3a-c, 4a-c, 6a, b, 7a-c). Cuticle thickness varies between species, approximately 1.2 μm (*P. socialis)*, 3.7 μm (*S. costarum)*, 2.5 μm (*G. oculata*), and 3 μm (*O. fusiformis*) (*n* = 20 measurements each). Numerous microvilli penetrate the cuticle; their density corresponds to that of the adjacent epidermal cells and forms a brush-border-like arrangement in *S. costarum* and *P. socialis* (Fig. 9e). The cuticle contains scarce fine fibrils forming a meshwork of similar density throughout its entire thickness. A distinct dense apical layer or epicuticle is absent in all species. The microvilli branch and surpass the cuticle proper by between approximately 1 μm in *P. socialis* and 0.5 μm in *G. oculata*. These microvilli originating from PSCs intermingle with those of receptor cells. Especially in the basal region, immediately above the apical cell membranes, these cell processes form an irregular dense network, apically overtopped by a layer comprising parallel densely arranged microvilli originating only from the PSCs (Fig. 7c, d). Finally, the microvilli terminate as an apical layer of densely arranged microvillar tips above the cuticle proper; in *G. oculata*, they appear as epicuticular projections. In most PSCs, a ciliary rootlet or a basal body giving rise to a short vestigial cilium was observed (e.g., Figs. 4b inset, 6c, 7c, d, 9e). These cilia do not extend above the cuticle and are shorter than those of the receptor cells. In PSCs with apical pigment-free extensions, cilia arise slightly above the pigment layer in *G. oculata* (Fig. 4b, inset). Another characteristic feature of the PSCs is their well-developed system of intermediate filaments (tonofilaments) (Figs. 4a, d, e, 6b, d, f, 7c, d, 8c, 9a-d). These form prominent bundles and are oriented in a basal-apical direction. Basally, the PSCs give rise to basal tube-like processes measuring 0.5–1.8 μm in diameter, which primarily contain these bundles of intermediate filaments (Figs. 6a, b, 8c, 9a, b). Upon reaching the ECM, hemidesmosomes are formed (Fig. 9b). Apically, the bundles split into somewhat smaller bundles, which also terminate in hemidesmosomes. These are situated in small and flat depressions of the apical membrane in the oweniids (Figs. 4d, e, 9c, e), whereas in *P. socialis* and *S. costarum*, the filaments branch into rather thin bundles entering the microvilli and finally form indistinct hemidesmosomes within these villi (Figs. 7d, 9c).

### The photoreceptor cells (PRCs)

The somata of the PRCs are situated below those of the PSCs (Figs. 3c, 5a, c, 6b, 7b, 8c, 9a, d). In *P. socialis*, the somata form clusters; each cluster is separated from others by tonofilament-containing processes of the PSCs (Fig. 8c), whereas in the oweniids, the cell bodies form a double layer with some overlap and are not that distinctly separated from those of PSCs (Figs. 3c, 5a, c, 6b). In all species, the somata located deepest are found immediately upon a basal network of neurite bundles forming the most basal part of the sensory epithelium (Fig. 3c). The somata are somewhat elongated, approximately 3.5–4 μm wide and 10–12 μm long, dominated by nuclei, which have a somewhat irregular outline in *G. oculata.* The somata contain numerous mitochondria, multivesicular bodies, and a well-developed endomembrane system. The latter comprises cisternae of rough and smooth endoplasmic reticulum and numerous clear vesicles also extending into the cell processes (e.g., Fig. 5a-f). From the cell bodies of the PRCs, thin processes extend apically and basally. The apical processes pass between the PSCs and terminate between the PSCs at the same level by forming apical junctions with their neighbors. Usually, the PRC processes are separated from each other and completely enclosed by PSCs. The processes of the PRCs are widest in *G. oculata and S. costarum,* whereas in *O. fusiformis* and *P. socialis*, they are thinner and somewhat inconspicuous. Especially in *O. fusiformis*, they may easily be overlooked. Diameters of apical processes range from 1.5–2.5 μm in *G. oculata* and 0.7–2 μm in *S. costarum* to 0.5–0.8 μm in *P. socialis* and 0.6 μm in *O. fusiformis*. In *G. oculata*, the processes of the PRCs only terminate between those PSCs possessing an apical pigment granule-free apical extension. Likewise, their junctional complexes, zonulae adherents, and septate junctions are situated at the same deeper level as in the adjacent cells. In contrast, in the other three species, no such situation was observed in the PRCs, and the junctions were located apically.

The PRC apices form a central depression, the depth of which depends on the level of the junctional complexes. Thus, it is most pronounced in *G. oculata*, which is up to 1.8 μm deep (Fig. 5b). At the base of this depression originates a cilium that extends into the subcuticular space and does not penetrate the cuticular and microvillar layers (Figs. 5a, b, d, 6e, 7c-e, 9c, f, h). The cilia rest on a basal body accompanied by an accessory centriole and a small inconspicuous rootlet (e.g., Fig. 5d, e). The length of the cilia could not be determined, but upon reaching the epithelial surface, they bend and lie horizontally above the adjacent cells (e.g., Fig. 6e, f, 7e, 9c, h). Depending on the species, more or less frequently sectioned cilia are observed in the apical layer of cell processes above the eye epithelium; these cilia are most numerous in *S. costarum* (Fig. 7c, d). The cilia are unbranched and very likely possess a typical 9 × 2 + 2 axoneme. In addition, the PRCs give rise to a couple of microvilli, which originate close to the base of the cilium and intermingle with those of the PSCs. The number and density of cell processes are higher in the chaetopterids, whereas in the oweniids, they appear comparatively low (e.g., Figs. 3c, 4b, c, 6b-f, 7b-d, 9c, h).

## Discussion

### Annelid phylogeny

Since the advent of phylogenomic data analyses, the backbone of the Annelid tree of life seems to be fairly robust, and most major groups are settled inside the tree [1, 6–9, 11, 24, 33], despite some initial criticism [34]. The majority of Annelida falls into a clade called Pleistoannelida, with Errantia and Sedentaria (including Clitellata) as the highest-ranked sister groups. In addition, at least three basally branching annelid clades were previously considered to belong to the errant or sedentary annelids (Fig. 1).

The basal grade includes Palaeoannelida with Oweniidae and Magelonidae, Chaetopteriformia with Apistobranchidae as the sister of Psammodrilidae and Chaetopteridae, and an unnamed clade comprising Amphinomidae, Euphrosinidae and Sipuncula (Fig. 1) [1, 7–11, 14, 33]. Previously, Magelonidae were usually grouped within Spionida [4, 35], and the phylogenetic position of Oweniidae was controversial. They were either regarded as being nested deeply within annelids close to Sabellidae and Siboglinidae [4, 35] or close to the annelid stem species [36–40]. Members of the clade Chaetopteriformia were thought not to be closely related but rather to be either within Spionida or Orbiniidae or close to Arenicolidae and Maldanidae [3, 4, 41–43]. The unexpected position and relationship of Sipuncula and Amphinomida are not corroborated by morphological apomorphies so far [16]. Amphinomida, comprising Amphinomidae and Euphrosinidae [44], are structurally similar to Errantia and were formerly considered to belong to this group based on morphological or molecular data (either as Errantia or Aciculata) [3, 6, 16, 35]. In contrast, Sipuncula is morphologically rather aberrant relative to typical annelids, lacking many of the so-called annelid key characters, such as segmentation and chaetae, and was considered not to be part of Annelida [1, 33]. Since their position, as indicated above, was constantly found in phylogenetic analyses, this new hypothesis is currently seen as the most probable [1, 7, 10, 11, 33].

Palaeoannelida and Chaetopteriformia especially bear several characteristics unusual for the majority of annelids, opening up a discussion about the ground pattern of Annelida, their synapomorphies, and character evolution within the taxon [6, 8, 25]. For instance, nuchal organs and the so-called rope ladder-like nervous system, formerly regarded to represent key characteristics of annelids, are absent in the two basal lineages [11, 16, 33]. These observations led to an ongoing series of studies focusing on these basal lineages and particularly their nervous system [11, 15, 16, 23, 24, 45–47]. With few exceptions, sensory organs were at most briefly mentioned or not considered in these studies. As a first result, according to Beckers and Tilic [16], nuchal organs most likely evolved in the stem lineage of Amphinomida + Sipuncula and Pleistoannelida.

With respect to the eyes of the ancestral annelid, a pair of bicellular eyes, i.e., the larval eyes, were usually considered to belong to the annelid ground pattern [8, 25]. However, the first appearance and evolutionary history of the adult eyes, their number, and their structure remained unresolved due to a lack of data in the basal lineages [20]. Within the two basal branches, larval eyes are usually present [48–50]. Large eyes, presumably representing adult eyes, have only been found in Oweniidae and Chaetopteridae. They are absent in Magelonidae, Apistobranchidae, and Psammodrilidae, as can also be confirmed by our observations [29, 31, 49, 51, 52]. The presence of adult eyes in Amphinomida and Sipuncula led to the hypothesis that adult eyes evolved at least in the stem lineage of Amphinomida + Sipuncula and Pleistoannelida [16, 20].

### The general structure of annelid eyes

Most annelids respond to light and possess some type of light-sensitive structure, which may comprise different types of PRCs, ciliary or rhabdomeric PRCs [17–19, 53]. However, only light-sensitive structures equipped with shading pigments, the structural prerequisite for detecting the direction of light, are generally called eyes, irrespective of whether they are capable of vision [54]; for a different definition, see [28]. The best feature of eyes is their remarkable diversity observable even in the single taxon Annelida [17, 18]. Generally, different types of eyes can be distinguished by their sequential occurrence during ontogeny and their presence on different body regions of these animals [17–19]. Among the latter, cerebral eyes located adjacent or within the brain are discerned from eyes present elsewhere on the body and generally regarded as homologous throughout Bilateria [26, 55–57].

Cerebral eyes are further differentiated into larval eyes and adult eyes to be distinguished by their molecular fingerprint, time of occurrence, fate during development, and often by structural features [19, 20, 53, 56, 57]. Larval eyes are those occurring first during ontogeny and are supplemented or replaced by the adult eye, often appearing only slightly later when the animal develops [56, 58, 59]. The fate of these larval eyes is not completely resolved; they may be replaced, persist beside the adult eyes, or may even be transformed into adult eyes depending on the taxon [56, 59–62]. However, structural distinction is often impossible if adult eyes are small and composed of only two or three cells forming an inverse pigment cup ocellus, which is the morphological signature of larval polychaete eyes [19, 56, 57, 63].

Generally, annelid adult eyes consist of two cell types, pigmented supportive cells (PSCs) and rhabdomeric photoreceptor cells (PRCs) (Fig. 1). These two cell types intermingle as a rule and build up a single-layered epithelium, the retina [19]. This epithelium typically forms a pigment cup into which the sensory processes of the PRCs project in all annelids studied to date [18, 19, 21, 22]. These PRCs are characterized by a considerable increase in their apical membrane surface, mostly in the form of highly ordered arrays of sensory microvilli [17–19]. These microvilli either arise from a more or less flattened apical PRC surface, or this apical surface is enlarged and forms a mushroom- or pillar-like extension, which enables the PRCs to bear even higher numbers of microvilli [17–19, 64]. Although conceivable for nondirectional and directional photoreception [27, 28], annelid eyes with PRCs lacking receptor membrane increases (or membrane stacking) are thus far unknown.

Additionally, PSCs may pass between the microvilli of the PRCs, extending into the eye cavity and finally forming a vitreous body or lens-like structure above the receptive processes (Fig. 1) [18, 19, 65]. Other cell types in certain species may also form lenses or vitreous bodies. Thus, the lumen of the eyecup is always completely filled with microvilli, vitreous bodies, or other light-guiding structures. Oriented towards the exterior, part of the epithelium forming the eyecup is devoid of shading pigment, allowing light access to the photosensitive microvilli from certain directions. Thus, depending on the width of the pupil or cup opening, in addition to simple pigment cup eyes, pinhole eyes or lens eyes may be present, allowing different modes of photoreception or even simple vision [27, 28]. In multiple cases, the lumen of the pigment cup is still continuous with the subcuticular space via a small canal lined by unpigmented supportive cells [20, 22, 58, 66–70]. This canal is indicative of the epidermal origin of the eyes during ontogeny. In contrast, in other species, the cavity is completely closed and separated from the exterior [18, 19, 22, 70, 71].

The number of cells and size of the eyes varies considerably among species. It ranges from only two cells, one of each type, to thousands of cells measuring less than 10 μm in diameter in certain meiofauna polychaetes (e.g., *Microphthalmus* spp. [see 64, 72]) to more than one millimeter in holoplanktonic annelids (e.g., *Vanadis formosa* Claparède, 1870 [see 17, 73]). Whereas bicellular eyes mostly have an inverse design, multicellular polychaete eyes are always converse (everse) in design; i.e., the light-sensitive processes project towards the incoming light [16–22, 70]. Very often, two pairs of such adult eyes are present, such as those usually occurring in Amphinomidae, Errantia (Phyllodocida and Eunicida), and in certain sedentaria. Therefore, two pairs of adult eyes are supposed to represent the basic pattern for a clade forming Amphinomida (together with Sipuncula) and Pleistoannelida [53]. Sometimes an additional pair of considerably smaller eyes, often composed of just two or very few cells, accompanies these two pairs of eyes [20–22, 70]. Mostly, it is unknown whether these small eyes represent persisting larval eyes or newly formed eyes.

In Sedentaria, large multicellular eyes of this type are rare and thus far only found in the comparatively basal sedentarian lineage Cirratulida (present in Flabelligeridae and Accrocirridae) [21, 74]. Smaller adult eyes comprising more than two cells are also present in Orbiniidae and Capitellidae [59, 62, 75]. However, usually in Sedentaria, adult eyes are reduced, bicellular, mostly inverse, and accordingly small [5, 76]. However, there are examples of such small cerebral eyes occurring in larger numbers, as well as the complete absence of eyes in Sedentaria. The ectopic eyes, especially present on the tentacles in fan worms or the median organ in Sabellariidae, are obviously secondarily evolved structures and, therefore, will not be considered here [76–80].

### Eyes of basal annelids

**Fig. 10:**
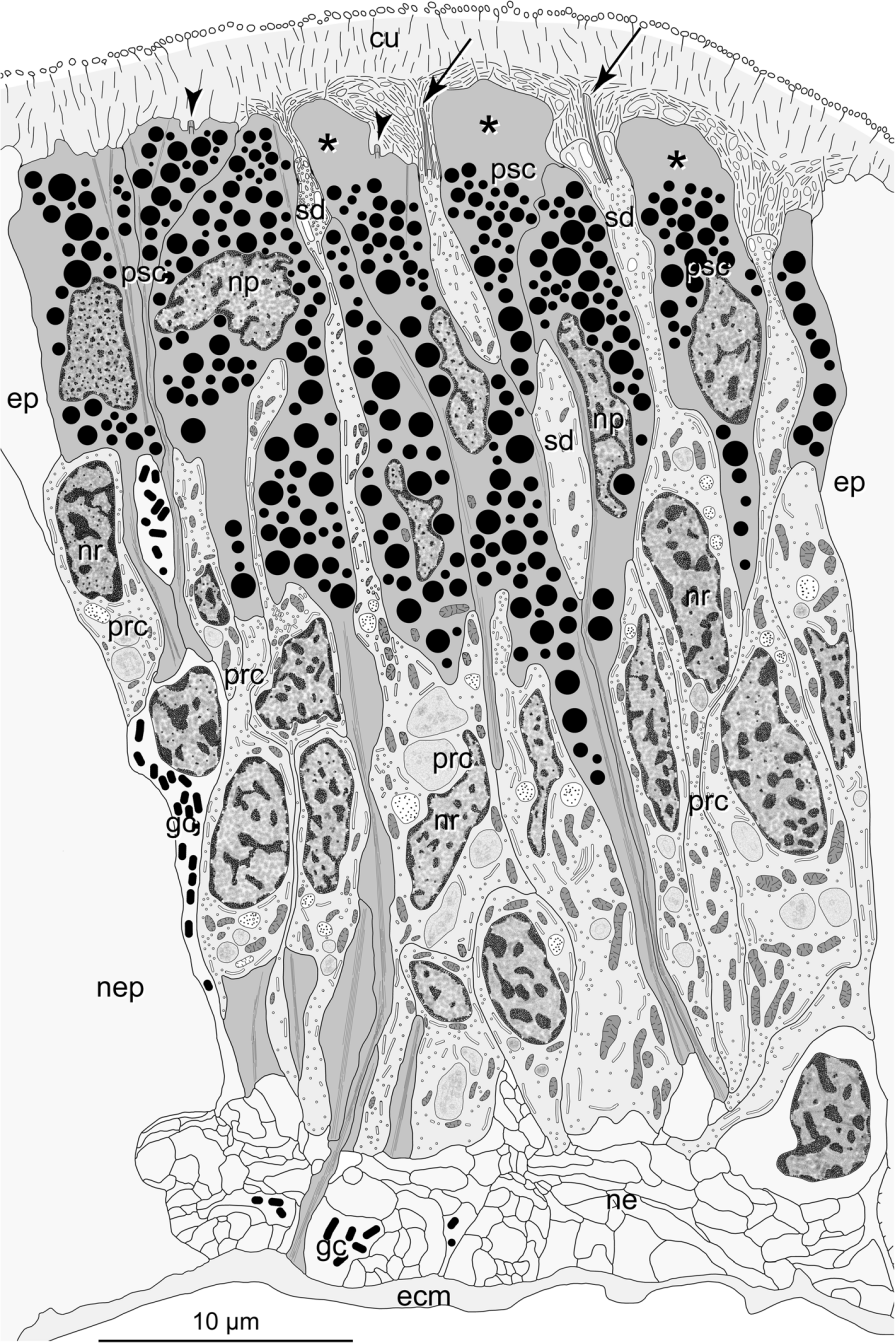
*Galathowenia oculata.* Reconstruction of cerebral eye exemplifying structural characteristics of cerebral eyes in basal Annelida, arrows: cilia of PRC, arrowheads: cilia of PSC, asterisks: pigment-free apical parts of PSC, epidermis and neuropil of the brain only indicated by light grey colour. *Abbreviations: cu cuticle, ecm ECM, ep epidermis, gc glial cell, ne neurite, nep brain neuropil, np nucleus of PSC, nr nucleus of PRC, prc photoreceptor cell, psc pigmented supportive cell, sd sensory dendrite of PRC*

Several differences and similarities become evident when comparing the eye structures observed in the basal lineages with those discussed above for Amphinomidae and Pleistoannelida (Fig. 10). First, eyes are not pigment cups rather than either eye spots (in Oweniidae) or eye pits with a retina restricted to one side only (in Chaetopteridae), which, in addition to the species investigated here, also applies to *Chaetopterus variopedatus* (Renier, 1804) [51]. Such types of adult eyes have thus far been unknown in annelids. The retinae are composed of two cell types, PSCs and PRCs, and the somata are found in similar positions as in Pleistoannelida. In particular, the eyes in the two oweniids studied, *O. fusiformis* and *G. oculata,* are not distinctly set off from the surrounding epidermis, which is not the case in Amphinomidae and Pleistoannelida. Whereas in *O. fusiformis*, the shading pigment is somewhat irregularly distributed in the eye region, it is concentrated apically in *G. oculata* and chaetopterids, as typical for annelid eyes. The arrangement of the two cell types is as found in other annelids: PSCs and PRCs intermingle, the latter sending only thin processes through the pigment layer (Fig. 10).

PRCs bear several unusual features, and most remarkable is their apical morphology, which differs only slightly from regular epidermal supportive cells. This observation means that the feature usually thought to represent one of the key characteristics of PRCs [17, 64], a considerable increase in the membrane surface, is absent or at most weakly developed. However, as Nilsson [28] emphasized, simple photoreception can be achieved by cells without morphological specializations, such as an increased sensitive membrane surface (increase in the number of cell processes or membrane stacking). However, the PRCs present in *G. oculata* might show an initial stage of membrane stacking. The PRCs possess a single cilium and a comparatively low number of microvilli, which may lead to the question of whether these PRCs are ciliary or rhabdomeric. Since a single cilium or a vestige thereof is common in rhabdomeric PRCs of Annelida [18–20, 22, 57, 70, 75], the presence of such a cilium is not ample criterion to distinguish PRCs from the respective opsins [26, 57, 61, 81–84]. For the time being, this problem cannot be resolved with certainty, but due to the other correspondences, we hypothesize that the PRCs of these basal annelids are of the rhabdomeric type. Antibody staining against opsins or gene expression studies may be applied to test this hypothesis.

According to Nilsson [27, 28], the eyes present in Oweniidae (Fig. 10) may be classified as simple eyes only capable of directional photoreception. The eyes found in Chaetopteridae and, in particular, those of *Spiochaetopterus costarum* show a more advanced structure, which exhibits an increase in the sensory membrane surface, allowing more advanced sensory tasks. Whether the pigment-free apices in the PSCs of *G. oculata* represent a light-guiding structure due to their lens-shaped appearance (see Fig. 3b) also remains speculative and unresolved. Due to or despite their simple structure, the eyes found in the basal annelid lineages are capable of various sensory tasks, such as circadian entrainment, depth measurement, UV detection, surface detection, phototaxis, or optical statocysts [27, 28]. These comparatively low-level photoreception capabilities correspond well to the more or less sessile and tube-dwelling lifestyle of these basal branching annelids. It may be noteworthy that the pygidial eyes found in *Paradialychone ecaudata* (Moore, 1923) (as *Chone ecaudata*) structurally resemble the cerebral eyes described for the basal lineages [85].

### Eyes of Sipuncula and Amphinomida

Concerning the eyes occurring in the two basal lineages, the eye structures present in the sister group of Pleistoannelida appear to be of special interest since this clade represents the branch adjacent to the taxa investigated in the present paper. In Amphinomida, comprising Amphinomidae and Euphrosinidae, there are generally two pairs of cerebral eyes, which are structurally similar to those present in Errantia [16, 22]. These are multicellular pigment cup eyes. In Amphinomidae, the eyes are connected to the exterior via a cuticularized canal. The so-called optical cavity is completely filled with sensory processes, but light-guiding structures such as vitreous bodies or lenses are absent [16, 22]. A remarkable feature is their wide pigment-free opening, which almost has the diameter of the entire optical cavity (e.g., Figs. 1, 2c in [22] and Fig. 7c in [16]). This opening may indicate their limited capability for vision, and they probably belong to low performing class III tasks, according to Nilsson [28]. This limitation contrasts with the eye described for the euphrosinid *Euphrosine foliosa* Audouin & H Milne Edwards, 1833, which possesses a highly differentiated shading pigment arrangement comprising isolated compartments of sensory microvilli and a lens [16]. This structural differentiation suggests higher vision capabilities than those found in its sister taxon but probably still low-resolution vision [28]. In summary, the eyes present in Amphinomida are structurally closer to those of Errantia than to those present in the more basal lineages.

In Sipuncula, the situation is different from that observed in Amphinomida: usually, there is only one pair of cerebral eyes in adults, each of which forms the basal part of an epidermal invagination called an ocular tube [86–89]. The eyes differ in structure between species, but in most cases, they represent invaginated pigment-spot eyes. In a few species, such as *Golfingia margaritacea* (Sars, 1851), a vesicular eye without an ocular tube is formed, whereas in others (*Sipunculus robustus* Keferstein, 1865 [as *S. angasi* Edmonds, 1955]), pigmentation is completely lacking [87, 88]. The ocular tubes emanate from the lateral sides of the cerebral organ, penetrating the brain capsule and extending into its anterior dorsal part [87, 88]. The epidermal cells of the cerebral organ and its cuticle continue into the tubes. Only in the basal part does the epithelium comprise PSCs and PRCs. The cuticle forms a plug-like structure and, in certain species, a dense structure called a refractive body. PSCs and PRCs intermingle, and the somata of the PRCs lie below those of the PSCs. The PRCs send a comparatively thin dendritic process apically, which gives rise to numerous microvilli and at least one cilium upon reaching the ocular tube. These cilia originate from a depression of the cell apex, extend between the disorderly arranged microvilli, and reach the cuticular plug lying above the cell processes. Thus, the adult eyes of Sipuncula are of the everse type comprising rhabdomeric PRCs, as generally found in Annelida. These eyes resemble the eyes present in members of the basal lineages, with the highest degree of correspondence found with Chaetopteridae.

In addition, sipunculans possess larval eyes, which comprise just a few cells: one PSC and two or three PRCs combined to form a minute eye of inverse design [87, 90, 91]. As in other annelids, these eyes develop quite early in ontogeny (e.g., 36 h after fertilization in *Golfingia vulgaris* (de Blainville, 1827)) [87, 92]. Later, in larger larvae at the end of the planktonic phase, an additional pair of eyes appears that can be assigned to the developing ocular tubes [87, 93]. In undetermined pelagosphera larvae, up to five pairs of eyes have been described [94]. However, the fate of these small eyes during further development and which of these represent the anlagen of the adult eyes is unknown. According to Åkesson [87], larval eyes very likely become reduced during or shortly after metamorphosis, and adult eyes are the sole eyes in adult Sipuncula, except for ectopic eyes present on the tentacles in *Sipunculus* spp. [87, 91].

## Conclusions

The morphological data on the cerebral adult eyes in members of the so-called basally branching lineages allow the presentation of a new and more complete picture of the evolution of eyes in Annelida. According to our data, a pair of adult cerebral eyes most likely also belongs to the ground pattern of the last common ancestor of annelids. Therefore, two generations of eyes, larval and adult eyes, must have been present in the annelid stem species [26] and, in all probability, expand the characters already discussed [8, 11, 15, 23, 25]. The members of the basal lineages Palaeoannelida and Chaetopteriformia, as well as Sipuncula in the next clade, are tube-dwelling or endobenthic organisms. Thus, it is not surprising that pigmented adult eyes may have been lost in a portion of their members.

These eyes are of rather simple structure with respect to both organ and cell structure (Fig. 10). For the first time, annelids possess pigment spot eyes with PRCs without or with only a moderate increase in the presumed photoreceptive membranes. In a second step, these pigment spots were internalized into tube-like invaginations, as present in Chaetopteridae and Sipuncula. This internalization coincided with a concentration of shading pigment in the PSCs and an increased membrane surface in the PRCs. These results corroborate the hypothesis of the evolution of PSCs and PRCs through stepwise segregation, change, and differentiation of the two cell types put forward by Arendt et al. [57].

Given that the backbone of the annelid tree represents the most probable phylogenetic scenario and that the sister group relationship of Sipuncula/Amphinomida remains stable and highly supported [8, 11, 13], pigment cup eyes must have been formed twice in annelids: in the lineage leading to Amphinomida and convergently in the pleistoannelid stem lineage (Fig. 1). These pigment cup eyes are further characterized by typical rhabdomeric PRCs with brush-border-like arrays of microvilli, which means that membrane stacking likewise must have occurred independently in both lineages. Moreover, as hypothesized by Randel and Jékely [26] for metazoans in general, simplicity in the ancestral structure of eyes can now be confirmed in the annelid stem species as well, probably only permitting photoreception and directional photoreception [27, 28]. Due to their simple structure and similarity to the regular epidermis in Oweniidae, Chaetopteridae, and Sipuncula, it is not surprising that eyes have not been detected in fossil annelids so far, although soft tissues are sometimes preserved [95, 96, and references in 26]. External invisibility, if investigated by scanning electron microscopy, also applies to many other annelids in which the epidermis and cuticle cover the eyes.

## Methods

### Material and collection

The study was performed with the oweniids *Galathowenia oculata* (Zachs, 1923) and *Owenia fusiformis* Delle Chiaje, 1844 and the chaetopterids *Phyllochaetopterus socialis* Claparède, 1869 and *Spiochaetopterus costarum* (Claparède, 1869). For comparison or proof of the absence of adult eyes, individuals of *Magelona mirabilis* (Johnston, 1865) (Magelonidae), *Chaetopterus norvegicus* M. Sars, 1835 (Chaetopteridae), *Apistobranchus tullbergi* (Théel, 1879) (Apistobranchidae), and *Psammodrilus balanoglossoides* Swedmark, 1952 (Psammodrilidae) were also included. Thus, the material investigated represents members from four out of five families comprising basal annelid radiation. Specimens of *G. oculata* were collected at the White Sea Biological Station (Kandalaksha Bay, Russia) in sublittoral zones by dredging by SV in 2016. *O. fusiformis* and *P. socialis* were collected intertidally in 2017 and 2018 near the Station Biologique Marine at Roscoff (Bretagne, France) (Ile Callot: *P. socialis,* St. Efflam: *O. fusiformis*).). In 2018, specimens of *S. costarum* and *M. mirabilis* were collected in the Anse de Poulduhan and *C. norvegicus* at the Point de Cabellou (Bretagne, France) during spring equinox low tide. Specimens of *P. balanoglossoides* were collected from a tidal flat near the Wattenmeerstation of the Alfred Wegener Institute at List/Sylt (North Sea, Germany) in spring 2018. Specimens of *A. tullbergi* (Théel, 1879) were collected at Quequertarsuaq, Disko Island, Greenland.

### Fixation and embedding

Tubes with animals were removed from the sediment, and animals were carefully removed from their tubes at the respective marine stations except for *Psammodrilus balanoglossoides,* which were directly washed out of sediment samples using the magnesium chloride technique [97]. Some specimens were used immediately for live observations under a dissecting or compound microscope. For electron microscopy, small adult individuals were chosen. They were relaxed for approximately 15 min in isotonic 8% magnesium chloride (MgCl_2_ × 6 H_2_O) with seawater immediately prior to fixation. Individuals of *Owenia fusiformis, Phyllochaetopterus socialis*, and *Psammodrilus balanoglossoides* were fixed in a solution of picric acid, paraformaldehyde, and glutaraldehyde (phosphate-buffered, 0.075 M) and adjusted to the appropriate osmolality with sucrose (SPAFG, [85]) for two hours at 4 °C. Specimens of *Galathowenia oculata* were fixed in 2.5% glutaraldehyde in Milling phosphate buffer [98] (pH 7.3–7.4; 2 × 1 h, RT). *Spiochaetopterus costarum* was fixed in 2.5% glutaraldehyde in 0.05 M phosphate buffer with 0.3 M NaCl. After initial fixation, the fixative was exchanged once. After five rinses in the appropriate buffer for 10 min each, specimens were stored in the same buffer containing 0.05% NaN_3_ at 4 °C until further processing.

Further processing was conducted in the zoology labs at Osnabrueck and Bonn universities. Specimens were postfixed in 1% OsO_4_ (phosphate-buffered, same buffer as above) for one hour at 4 °C. After being washed for 5 min in either 0.075 M buffer adjusted with sucrose or a 0.05 M buffer adjusted with NaCl samples that were dehydrated using an ethanol series (30% for five minutes at 4 °C, 50% for five minutes at 4 °C, 70% for 10 min at 4 °C, 80% for 10 min at 4 °C, 95% for 10 min at 4 °C, 95% for 10 min RT, 2 × 100%, 10 min RT). Specimens chosen for TEM and light microscopy were then dissected into smaller parts. Only the anterior ends were further processed. These were transferred into a solution of ethanol and intermediate propylene oxide (100% ethanol:propylene oxide, 1:1, 2 × 30 minutes), followed by pure propylene oxide (4 × 15 minutes). This solution was replaced by mixtures of the intermedium and the embedding medium, starting with propylene oxide: Araldite/Epon (PolyBed 812) 3:1 for six hours, followed by 2:1 (12 h) and finally 1:1 (12 h). The intermedium was then allowed to evaporate overnight. Before final embedding took place, specimens were transferred into drops of fresh Araldite/Epon for 5 min at 60 °C. After two repetitions, specimens were brought into the embedding molds. Polymerization was carried out at 60 °C for 72 h.

### Sectioning and microscopy

Specimens were cut into a series of semithin sections (1 μm) using diamond knives (Diatome, Biel, Switzerland) and UC6 or UC7 Leica ultramicrotomes (Wetzlar, Germany). After the eyes were found, a combination of semithin and ultrathin sections (70 nm) was cut by taking a short series of ultrathin sections (approximately 20–40 sections) every 5 μm until the eye region was cut, except for *Psammodrilus balanoglossoides*, for which a complete series of ultrathin sections of the anterior end was obtained. Ultrathin sections were placed on single-slot grids coated with pioloform support films. Then, they were contrasted at 20 °C with 2% uranyl acetate (30 min) and 0.5% lead citrate (20 min) in a Nanofilm Surface Analysis Ultrastainer® (Göttingen, Germany). Finally, the sections were examined with Zeiss® EM 902A and Zeiss Libra 120 transmission electron microscopes (Oberkochen, Germany). Images were recorded using CCD cameras (Image SP®, 4 k, Mohrenweis, Germany). Semithin sections were collected on glass slides, stained with toluidine blue (0.5% toluidine blue in a 1% aqueous solution of borax for 15–30 s at 60 °C), rinsed with H_2_O, fixed with 5% ammonium molybdate tetrahydrate ((NH4)6Mo7O24 × 4 H2O) and mounted with Entellan mounting medium, except for sections of *Galathowenia oculata* in which the staining remained unfixed. Pictures were taken with a DMLS light microscope (Leica, Wetzlar, Germany) equipped with a Progress Gryphax® CCD camera (Jenoptik, Jena, Germany) and Gryphax software.

## Data Availability

The material of *O. fusiformis*, *P. socialis*, and *P. balanoglossoides* (embedded blocks, semithin sections, and ultrathin sections) is stored at the Department of Zoology and Developmental Biology at the University of Osnabrueck. All images taken are stored in the database Omero hosted at the University of Osnabrueck. These images are available from the corresponding author upon reasonable request. The respective material of *G. oculata* is stored in the Department of Invertebrate Zoology, Biological Faculty, Lomonosov Moscow State University, Moscow. Materials of *M. mirabilis, C. norvegicus*, *S. costarum*, and *A. tullbergi* (embedded blocks, semithin sections, and ultrathin sections) were stored at the Institute of Evolutionary Biology and Ecology at the University of Bonn.

## Abbreviations

bb: Basal body
br: Brain
ce: Accessory centriole
ci: Cilium of PSC
coe: Coelom
cr: Ciliary rootlet
cu: Cuticle
cui: Cuticular invagination
dbv: Dorsal blood vessel
ecm: ECM
ECM: Extracellular matrix
ep: Epidermis
ey: Eye
g: Gut
gl: Glandular cell
if: Intermediate filaments
LM: Lightmicrocopy
lbv: Lateral blood vessel
m: Mitochondrion
mu: Muscle fiber
mv: Microvillus
n: Nucleus
ne: Neurite
nep: Neuropil
np: Nucleus of PSC
nr: Nucleus of PRC
pa: Palp
per: Peristomium
pg: Pigment granule
ph: Pharynx
PRC: Photoreceptor cell
prc: PRC
pro: Prostomium
PSC: Pigmented supportive cell
psc: PSC
pt: Peritoneum
sc: Sensory cilium
sd: Sensory dendrite
smv: Sensory microvilli
TEM: Transmission electronmicroscopy
v: Vesicle
za: Zonula adherens
